# Treating disease progression with osimertinib in EGFR-mutated non-small-cell lung cancer: novel targeted agents and combination strategies

**DOI:** 10.1016/j.esmoop.2021.100280

**Published:** 2021-10-09

**Authors:** V. Di Noia, A. D’Aveni, E. D’Argento, S. Rossi, P. Ghirardelli, L. Bortolotti, V. Vavassori, E. Bria, G.L. Ceresoli

**Affiliations:** 1 Medical Oncology 1 Unit, IRCCS Regina Elena National Cancer Institute, Rome, Italy; 2 Department of Medical Oncology 1, Cliniche Humanitas Gavazzeni, Bergamo, Italy; 3 Comprehensive Cancer Center, Fondazione Policlinico Universitario Agostino Gemelli IRCCS, Rome, Italy; 4 Department of Oncology and Hematology, Humanitas Clinical and Research Center, Rozzano, Italy; 5 Istituto di Medicina Interna e Geriatria, Università; Cattolica del Sacro Cuore, Rome, Italy

**Keywords:** EGFR, osimertinib, non-small-cell lung cancer, tyrosine kinase inhibitors, progression

## Abstract

A precision medicine approach has been successfully applied in medical oncology for the treatment of non-small-cell lung cancer (NSCLC) through the identification of targetable driver molecular aberrations; activating mutations of epidermal growth factor receptor (EGFR) are the most common. Osimertinib, a third-generation, wild-type sparing, irreversible EGFR tyrosine kinase inhibitor (TKI), originally showed a striking activity after progression to first- and second-generation EGFR-TKIs when T790M resistance mutation was identified. Thereafter, upfront use of osimertinib became the standard of care based on overall survival benefit over first-generation TKIs erlotinib and gefitinib as reported in the FLAURA trial. For patients progressing on osimertinib, identification of resistance mechanisms is crucial to develop novel targeted therapeutic approaches. Moreover, innovative drugs or combination therapies are being developed for cases in which a specific resistance mechanism is not identifiable. In this review, the post-osimertinib treatment options for EGFR-mutated NSCLC are analyzed, with an outlook to ongoing clinical trials. An algorithm to guide clinicians in managing progression on osimertinib is proposed.

## Highlights

• Acquired resistance mechanisms to osimertinib can be EGFR dependent or independent.
• First- and fourth-generation EGFR-TKIs are able to overcome EGFR-dependent resistance.
• MET and MEK inhibitors may overcome EGFR-independent resistance.
• Anti-HER/MET novel monoclonal antibody seems effective across multiple resistance mechanisms.
• Carboplatin/paclitaxel ± bevacizumab/atezolizumab represents a feasible option in ‘fit’ patients progressing on osimertinib.

## Introduction

A precision medicine approach has been successfully applied in medical oncology for the treatment of non-small-cell lung cancer (NSCLC) through the identification of targetable driver molecular aberrations; activating mutations of epidermal growth factor receptor (*EGFR*) are the most common (10%-15% of Caucasian and 30%-40% of Asian patients with non-squamous histotype).^1^ Nowadays, three generations of EGFR tyrosine kinase inhibitors (TKIs) are available: first-generation reversible EGFR inhibitors, erlotinib and gefitinib; second-generation irreversible EGFR family blockers, afatinib and dacomitinib; and a third-generation, wild-type sparing, irreversible EGFR inhibitor, osimertinib. Erlotinib, gefitinib, and afatinib improved the objective response (range, 65%-90%) and progression-free survival (PFS) (range, 9-14.7 months) compared with platinum-based chemotherapy in a first-line setting.^2^, ^3^, ^4^, ^5^, ^6^, ^7^, ^8^, ^9^, ^10^ Overall survival (OS) benefit was demonstrated for both dacomitinib, in the phase III ARCHER 1050 trial (versus gefitinib),^11^ and osimertinib, in the phase III FLAURA study (versus gefitinib and erlotinib).^12^^,^^13^ The more tolerable toxicity profile and activity against brain metastases favor the use of osimertinib over dacomitinib as first-line treatment. Osimertinib originally demonstrated striking activity after progression to first- or second-generation TKIs compared with platinum doublets (in the phase III AURA3 trial) when the *EGFR* resistance mutation T790M was detected (50%-63% of cases).^14^

Recently, osimertinib demonstrated to dramatically improve disease-free survival (DFS) compared to placebo as adjuvant therapy in resected stage IB-IIIA EGFR-mutated NSCLC (ADAURA trial).^15^ This relevant finding raised interesting open questions, such as the translation of DFS benefit in a more relevant OS advantage and the potential efficacy of re-challenge therapy with osimertinib in patients who experienced a late relapse of disease. Another clinical issue under investigation in the ADAURA trial is the clinical utility of liquid biopsy including circulating tumor DNA (ctDNA) analysis in the monitoring of the behavior of the disease in the adjuvant setting.

Regarding the metastatic setting, a key question is whether it is best to use osimertinib upfront or retain it for second-line use after the failure of a previous EGFR-TKI. There is some evidence of prolonged OS achieved with a sequential TKI approach,^16^ such as emerged for the sequence afatinib–osimertinib in the retrospective GioTag study.^17^ However, only a minority of patients (about one-third across the trials or in real life) receive subsequent osimertinib, reflecting the challenging identification of T790M resistance mutation, which often requires a re-biopsy even though liquid biopsy techniques and next-generation sequencing (NGS) assays can facilitate the detection.^18^^,^^19^ Furthermore, osimertinib showed to prolong survival outcome irrespective of the presence of brain metastases and additionally prevented the occurrence of central nervous system (CNS) disease progression, which represented a common event in *EGFR*-mutated NSCLC.^20^ The relevant CNS activity of osimertinib demonstrated in the phase III AURA3 and FLAURA trials is another point of strength of this third-generation TKI compared with the first- and second-generation TKIs, which are characterized by poorer CNS penetrance.

**Figure 1:**
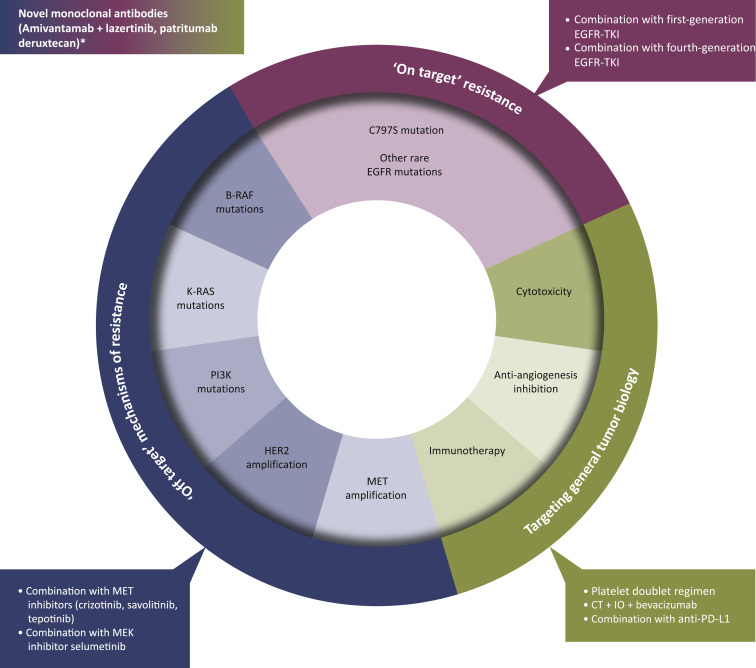
Mechanisms of resistance to osimetinib and potential strategies of treatments to overcome resistance. CT, chemotherapy; IO, immunotherapy. ∗Activity demonstrated across resistance mechanisms.

Nevertheless, the use of osimertinib upfront is encouraged by the continuous introduction of novel targeted therapy directed against druggable resistance mechanisms and by the development of innovative treatment strategies for disease progression not related to a specific molecular mechanism. Here, we review the post-osimertinib treatment options for *EGFR*-mutated NSCLC (Figure 1), providing an overview of ongoing clinical trials (Table 1) and proposing an algorithm to guide clinicians in managing progression on osimertinib.

**Table 1:** Ongoing clinical trials on acquired resistance to osimertinib in EGFR-mutated NSCLC

Phase	Clinical trial number	Drug(s) class	NSCLC trial Population	Line of treatment	Treatment arm(s)	Primary endpoint	Status
III	NCT03515837 (KEYNOTE 789)	Combination of PD-1 inhibitor with CT	EGFR mutated	2-3	Experimental: Pembrolizumab + pemetrexed + chemo Active comparator: Placebo + pemetrexed + chemo	- PFS, OS	Recruiting
II	NCT03778229 (SAVANNAH)	MET inhibitors	EGFR mutated with MET amplification/high expression	2 ≤ *n* ≤ 4	Osimertinib + savolitinib	- ORR	Recruiting
II	NCT03944772 (ORCHARD)	MET inhibitors, first-generation anti-EGFR-TKI, anti-EGFR mAbs, combination of CT plus anti-PD-L1 mAbs	EGFR mutated	2	Osimertinib + savolitinib Osimertinib + gefitinib Osimertinib + necitumumab Durvalumab + carboplatin + pemetrexed	- ORR	Recruiting
II	NCT03940703 (INSIGHT-2)	MET inhibitor	EGFR mutated with MET amplification	≥1	Tepotinib and osimertinib	- Safety - ORR	Recruiting
I/II	NCT03784599 (TRAEMOS)	Anti-HER2-conjugated antibody	EGFR-mutated NSCLC and HER2 amplification or high expression	≥2	Trastuzumab–emtansine and osimertinib	- Safety - ORR	Recruiting
Ib	NCT04001777	Bcl-2 family protein inhibitor	EGFR-mutated third-generation TKI resistant or treatment naive	Any lines	APG-1252 plus osimertinib	- MTD - RP2D	Recruiting
I	NCT03891615	PARP inhibitor	EGFR mutated	≥2	Osimertinib + niraparib	- MTD	Recruiting
Phase I	NCT03516214 (EATON)	Third-generation anti-EGFR-TKI, MEK inhibitor	EGFR mutated, including TKI naive	Any lines	Nazartinib and trametinib	- MTD - RP2D	Recruiting
Phase II	NCT02759835	—	EGFR mut oligoprogressive disease (no more than five sites of progressive disease)	≥1	Osimertinib followed by LAT followed by osimertinib LAT followed by osimertinib	- PFS2	Active, not recruiting
II	NCT04136535 (ALTER-L031)	Multitarget TKI	EGFR mutated	≥1	Pemetrexed and carboplatin with or without anlotinib	- PFS	Active not yet recruiting
II	NCT03532698	NSAID	EGFR mutated	2	Osimertinib + aspirin	- ORR	Not yet recruiting
II	NCT04316351	Anti-PD-1 mAb, multitarget TKI	EGFR mutated with T790M	≥3	Toripalimab + pemetrexed + anlotinib	- ORR	Not yet recruiting

AE, adverse events; CT, chemotherapy; EGFR, epidermal growth factor receptor; LAT, locally ablative therapy; mAb, monoclonal antibody; MDT, maximum tolerated dose; NSAID, nonsteroidal anti-inflammatory drug; NSCLC, non-small-cell lung cancer; ORR, overall response rate; OS, overall survival; PARP, poly(ADP-ribose) polymerase; PFS, progression-free survival; RP2D, recommended phase II dose; RR, response rate; TTP, time to progression.

## Identified mechanisms of resistance to osimertinib

Resistance invariably occurs against osimertinib in front-line therapy as well as after previous TKI.^21^ Identification of the resistance mechanisms is crucial to develop novel targeted therapeutic approaches. Resistance mechanisms can be EGFR dependent or ‘on-target’ and EGFR independent or ‘off-target’. In the first case, tumor cell proliferation continues to depend directly on EGFR signaling. Off-target resistance is characterized by the predominance of other parallel molecular pathways that bypass EGFR signaling.^22^ Relevant data have emerged from plasma analysis of ctDNA by NGS in patients who progressed on osimertinib therapy in the FLAURA and AURA3 studies.^23^^,^^24^ Although resistance mechanisms to osimertinib appear to be similar regardless of whether it is used in a first- or second-line setting, resistance to front-line osimertinib may be even more reliant on off-target pathways than resistance to later-line osimertinib, in which cancers have already shown dependence on EGFR through T790M.

In FLAURA, 91 patients were evaluated, identifying *MET* amplification (15% of cases) and the tertiary *EGFR* resistance mutation C797S (7% of patients) as the most frequent resistance mechanisms. In line with pharmacodynamics of osimertinib, no evidence of acquired T790M was observed. Other mechanisms included the emergence of *PIK3CA* (7%), *BRAF* (3%) or *KRAS* mutations (3%), human epidermal growth factor receptor 2 (HER2) amplification (2%), and other rare *EGFR* secondary mutations.^23^ The analysis of 73 patients enrolled in the AURA3 study, who were all T790M positive and received osimertinib in second line, revealed MET amplification (19%) and emergence of C797S (15%) as the most common resistance mechanisms, followed by cell-cycle gene alterations (12%), *HER2* amplification (5%), and *PIK3CA* amplification/gene alterations (5%). Of note, 19% of patients had more than one putative resistance mechanism and loss of T790M was observed in 49% of cases. All patients with acquired tertiary EGFR mutations retained the T790M mutation after progression on osimertinib.^24^

In another study including 41 patients treated with second-line osimertinib, NGS of tumor biopsies detected loss of T790M in 68% of samples tested in association with a range of competing resistance mechanisms, such as *KRAS* mutations and targetable gene fusions. Interestingly, time to treatment discontinuation was shorter in patients with T790M loss (6.1 versus 15.2 months), suggesting emergence of pre-existing resistant clones, and a small-cell lung cancer (SCLC) transformation was identified in ∼21% of patients.^25^

NGS technology can help to obtain a baseline genetic portrait of *EGFR*-mutated tumors which may allow for the discovering of additional concurrent mutations that may be responsible of primary resistance to EGFR-TKI. In fact, concurrent genetic mutation of *TP-53* and other genes such as *KRAS*, *CTNNB1*, *PIK3CA*, *SMAD4*, and *MET* were found to be related to worse outcomes of first-line gefitinib.^26^ The role of concurrent mutations in driving and predicting primary resistance for osimertinib needs to be further explored. In these cases of resistance sustained by a complex genetic scenario, combination strategy of chemotherapy with targeted agents may have a biological rationale. The FLAURA-2 phase III trial, which is exploring the combination of osimertinib with platinum doublets as first-line treatment in comparison with osimertinib single-agent therapy, will provide relevant information on the potential effect of combination treatment in delaying the occurrence of TKI resistance.

The importance of the histological transformation as an acquired resistance mechanism to osimertinib was confirmed in another recent study in which analysis of tumor samples of 71 patients found small-cell or squamous histotype transformation in 14% of cases overall and 19% of samples from patients treated with first-line osimertinib.^27^ Recently, Belluomini et al. reported a case of histological transformation to large-cell neuroendocrine carcinoma as resistance mechanism to osimertinib.^28^ NGS analysis of tumor tissue at diagnosis and when disease progression occurred revealed the presence since the diagnosis of the molecular alterations, such as *TP53* and *RB1* inactivation, which are often associated to histological transformation in high-grade neuroendocrine carcinoma. These findings may support the early use of NGS to identify the cases of primary resistance mediated by pre-existing subclones which may derive more benefit from a combination treatment with chemotherapy and EGFR-TKI.

Although tissue biopsy could overcome some of the limits of plasma genotyping, including suboptimal detection of lineage plasticity, copy number changes, and chromosomal rearrangements, no clear mechanism of resistance is identified in a relevant proportion of patients treated with osimertinib (40%-60% across lines of therapy). Epigenetic modifications or changes in protein expression may play a relevant role.

In particular, the transcriptional dysregulation which causes the activation of the yes-associated protein (YAP) and the forkhead box protein M1 axis has been identified as driver of epithelial–mesenchymal transition (EMT)-associated EGFR-TKI resistance. Furthermore, high YAP activity seems to lead the evasion of TKI-induced apoptosis through the repression of the pro-apoptotic protein Bcl2 Modifying Factor.^29^, ^30^, ^31^

## Therapeutic strategies for progression to osimertinib

### Overcoming on-target resistance

#### First-generation EGFR-TKIs

The most common tertiary *EGFR* mutation is EGFR C797S, which accounts for 10%-26% of cases of resistance to second-line osimertinib and represents the second most frequent mechanism of resistance (7% of cases) behind *MET* amplification when osimertinib is administered in first line.^23^^,^^24^ The EGFR C797S mutation, in which cysteine at codon 797 within the ATP-binding site is substituted by serine, prevents the covalent bond between osimertinib and the mutant *EGFR*, resulting in drug resistance.^32^ Preclinical findings demonstrated that the efficacy of first- and second-generation TKI is not affected by the cysteine at position 797, thus suggesting that treatment with these drugs might be a strategy to overcome *EGFR* C797S resistance mutation acquired following osimertinib.^33^^,^^34^ However, due to the concurrent T790M mutation in patients who developed resistance to osimertinib after the failure of previous first- or second-generation inhibitors, a combinatorial treatment with osimertinib and a first- or second-generation TKI is required to overcome resistance.^35^ In this context, the configuration of the T790M and C797S mutations affects how tumor cells could respond to therapy. When the two mutations are on different alleles (*in trans*), the combination of first- and third-generation TKIs can restore EGFR inhibition. To date, clinical proof of efficacy of this combinatorial strategy is limited to case reports.^36^ The ongoing ORCHARD trial includes the evaluation of a cohort of patients receiving osimertinib with gefitinib after the development of C797S-dependent resistance to osimertinib.^37^ Conversely, the presence of the two mutations on the same allele (*in cis*) confers resistance to all generations of EGFR-TKI, thereby suggesting the need for alternative treatment strategies.^35^ Brigatinib, best known as ALK inhibitor but originally developed as a dual inhibitor of EGFR and ALK, in combination with cetuximab showed capability of overcoming concomitant C797S and T790M *in cis* in a preclinical study.^38^^,^^39^ A case report of resistance to first-line osimertinib in advanced NSCLC confirmed that this combination was active in this setting.^39^

The development of C797S in the absence of the T790M mutation, as occurs when osimertinib is used upfront, confers resistance to third-generation EGFR-TKI while sensitivity to first-generation TKI is retained.^40^ Erlotinib and, to some extent, gefitinib were active against the activating EGFR mutations and the C797S mutation.^41^^,^^42^ It has been also hypothesized that combining first- and third-generation EGFR-TKIs may delay the onset of the C797S and T790M resistance mutations, given the efficacy of each agent against these respective mutations. Clinical trials investigating the combination therapy in this context are currently ongoing.

Besides C797S mutation, other rare *EGFR* mutations were described as conferring osimertinib resistance. Mutations in G796 (G796R, G796S, and G796D), L792 (L792H), L718 (L718Q), G719 (G719A), and G724 (G724S) have been identified, and based on protein structure prediction, they can sterically interfere with the binding of osimertinib to the EGFR kinase domain. *In vitro* studies demonstrated that these rare mutations might still be sensitive to first- and second-generation EGFR-TKI.^43^, ^44^, ^45^, ^46^, ^47^, ^48^, ^49^, ^50^, ^51^ These findings have to be confirmed in the clinical setting.

#### Fourth-generation EGFR-TKIs

Another strategy to overcome C797S-dependent resistance consists of the development of fourth-generation TKIs able to inhibit both C797S and T790M signaling.^52^ So far, EAI045 is the first allosteric TKI engineered for this purpose. Mutations in C797S do not seem to affect the efficacy of EAI045 because its allosteric binding pocket is not influenced by this cysteine residue. However, EGFR receptor dimerization makes the inhibition mediated by the drug alone ineffective. The activity against T790M and C797S could be restored by the combination with cetuximab.^53^ JBJ-04-125-02, another fourth-generation EGFR-TKI, has recently been found to be active against EGFR C797S-T790M-L858R signaling *in vitro* and *in vivo*, and the combination of JBJ-04-125-02 with osimertinib was more effective than either single agent alone.^54^ The clinical efficacy of these novel TKIs has still to be tested.

### Targeting off-target resistance

#### MET inhibitors

*MET* amplification is one of the most frequent mechanisms of acquired resistance to osimertinib, occurring with a prevalence of 15% and 19% according to the use of osimertinib in first or subsequent lines of therapy, respectively.^23^^,^^24^ *MET* amplification-dependent resistance is caused by a persistent activation of signaling pathways downstream of EGFR, such as those mediated by mitogen-activated protein kinase (MAPK), signal transduction and activator of transcription, and phosphatidylinositol 3-kinase-Akt, which bypass EGFR activation and signaling.^55^

Several preclinical studies have demonstrated that the concomitant use of MET inhibitors with osimertinib has the potential to overcome resistance in osimertinib-resistant *EGFR*-mutant NSCLC cell lines with MET gene amplification.^56^, ^57^, ^58^ Clinical experience in two patients suggested that combination of crizotinib, an ALK inhibitor with anti-MET activity, with osimertinib or erlotinib might overcome MET-mediated resistance.^59^, ^60^, ^61^

Savolitinib is an oral, potent, and highly selective MET TKI, investigated in combination with osimertinib in the phase Ib TATTON trial, exploring different osimertinib combinations according to the acquired resistance mechanisms. Part B of the trial enrolled patients with MET-amplified, EGFR mutation-positive NSCLC who had progressed on EGFR-TKI, including a third-generation EGFR-TKI (cohort B1) or an earlier-generation EGFR-TKI with the presence (cohort B3) or absence (cohort B2) of T790M resistance mutation. Osimertinib was given at 80 mg and savolitinib at 600 mg daily. In the overall population of part B, the most common adverse events of grade ≥3 were an increase in aspartate aminotransferase (7%) and a decrease in neutrophil count (7%). Serious adverse events were reported in 45% of patients with the most common being anaphylactic reaction (4%) and pneumothorax (4%). Two deaths occurred due to acute renal failure and an unknown cause and were considered potentially related to the treatment. In the cohort of 69 patients who had received previous first- or second-generation EGFR-TKI, treatment with osimertinib plus savolitinib yielded an overall response rate (RR) of 30%, whereas among the 18 MET-positive patients with disease progression following osimertinib, the overall RR was 67%, with a median duration of response of 12.4 months.^62^ The lower RR observed in subcohort B1 (resistance to osimertinib) compared with subcohort B2 and B3 (resistance to first- or second-generation TKI) could be in part related to the inclusion of more heavily pretreated patients in subcohort B1, who received osimertinib as first line in a few cases only because of the timing of TATTON trial.

Based on the acceptable risk–benefit profile and encouraging antitumor activity, the specific sequence of first-line osimertinib, followed by osimertinib and savolitinib, requires further study and is being investigated prospectively in the SAVANNAH trial (NCT03778229).

Tepotinib, another oral selective MET TKI, in combination with osimertinib is still under investigation in the INSIGH2 trial (NCT03940703), a single-arm phase II study enrolling patients with advanced NSCLC with resistance to first- to third-generation EGFR-TKIs driven by MET amplification.^63^

#### MEK inhibitors

Another acquired resistance mechanism to EGFR-TKIs is upregulation of the RAS/RAF/MEK/ERK signaling pathway, key to cell survival and proliferation; this can occur downstream of various other signaling pathways. Selumetinib is an inhibitor of mitogen-activated protein kinase (MEK or MAPK/ERK kinase) 1 and 2. The combination of osimertinib with selumetinib has been evaluated in the TATTON trial. In the dose-finding part (part A), different schedules of intermittent or uninterrupted selumetinib were evaluated. The most prevalent treatment-related adverse events included diarrhea (75%), rash (58%), and nausea (47%). Intermittent dosing appeared more tolerable than continuous administration, with no dose-limiting toxicities, thus the 75-mg twice daily on a 4 days-on/3 days-off schedule was chosen for the dose expansion part (part B). The overall RR was 43% in part A (36 patients enrolled), while in part B (47 patients) the partial response was 34%. After receiving a third-generation TKI as first-line treatment, the combination was able to yield a partial RR of 17% in part A of the study and 23% in part B, even though these are observations from relatively small numbers.^64^^,^^65^

#### Novel monoclonal antibodies

Phase I studies have been ongoing to evaluate novel monoclonal antibodies (mAbs) in EGFR mutation-positive NSCLC, including patients with acquired resistance to osimertinib.^66^^,^^67^

Amivantamab is a fully humanized, bispecific immunoglobulin G1 antibody, directed against both EGFR and MET receptor, which was shown to block ligand binding, promote receptor degradation, and trigger antibody-dependent cellular cytotoxicity in models of EGFR mutation-positive NSCLC.^68^ Amivantamab has been recently approved by the Food and Drug Administration for the front-line treatment of adult patients with *EGFR* exon 20 insertion-mutant NSCLC.^69^

CHRYSALIS (NCT02609776) is an open-label, multicenter, first-in-human study to evaluate the safety, pharmacokinetics, and preliminary activity of amivantamab as a monotherapy and in combination including lazertinib, a novel third-generation anti-EGFR-TKI. Results of the expansion cohort of 45 patients with osimertinib-relapsed, chemotherapy-naive disease have been recently presented at the ASCO2021 Virtual Meeting.^66^ After a median follow-up of 8.2 months, an overall RR of 36% and a median PFS of 4.9 months were observed in the whole expansion cohort. The safety profile was manageable and consistent with EGFR and MET inhibition, including infusion-related reaction (78%), rash (51%), paronychia (49%), and constipation (22%) as the most frequent adverse events, most of which were grade 1-2. The analysis of resistance mechanisms by NGS of tumor tissue and ctDNA revealed a not-negligible activity of the combination both in the EGFR-MET-mediated subgroup (RR = 47%) and in the group of patients with unknown resistance mechanisms (RR = 50%), while no responses were recorded in case of EGFR-MET-independent resistance. Nevertheless, the immunohistochemistry (IHC) analysis of tissue for EGFR and MET expression showed an RR of 90% among patients (10/20) having high expression of both receptors (combined EGFR + MET H score >400), of whom five patients were without an unidentified resistance mechanism by NGS. This finding suggests a potential usefulness of IHC evaluation for selecting patients who may derive the most benefit from the combination therapy, although further confirmation is needed.^70^

Patritumab deruxtecan is a human anti-human epidermal growth factor receptor 3 (HER3) antibody attached to a novel topoisomerase I inhibitor payload by a tetrapeptide-based linker. HER3 expression is also associated with increased metastases and reduced survival in patients with NSCLC, where frequency has been reported to be as high as 75%.^71^ An ongoing multicenter phase I trial is assessing the safety/tolerability and preliminary activity of patritumab deruxtecan in patients with advanced *EGFR*-mutated NSCLC who are pretreated with chemotherapy and develop disease progression on osimertinib or were T790M negative after disease progression while on erlotinib, gefitinib, or afatinib.^67^ The median number of lines of therapy received was 4. Safety data on 57 patients showed a manageable profile. The most common treatment-emergent adverse events of grade 3 included thrombocytopenia (30%), neutropenia (19%), and fatigue (14%). The discontinuation rate for toxicity is 11%. Drug-related interstitial lung disease occurred in 4% of patients and was non-fatal in all cases. Notably, in the 44 patients who had received osimertinib, the RR was 39%. Responses were observed across the mechanisms of resistance and in the cases without an identified mechanism. Furthermore, the drug seemed to be active irrespective of the level of IHC HER-3 expression.

Moreover, the safety and activity of patritumab deruxtecan in patients with advanced *EGFR*-mutated NSCLC after failure of EGFR-TKI and platinum-based chemotherapy have been evaluated in a phase I clinical trial (NCT03260491). Fifty-seven patients have been enrolled. Results showed promising evidence of preliminary antitumor activity and safety due to the administration of 5.6 mg/kg of patritumab deruxtecan.^72^

These novel mAbs could be a promising therapeutic approach for patients with *EGFR*-mutated NSCLC, with the advantage of potential activity on various mechanisms of resistance to EGFR-TKI. Obviously, more data on the survival impact of these treatments are largely awaited from the further experimental phases.

### Other ongoing clinical trials

Other phase I/II studies are currently ongoing to assess the safety and clinical activity of novel combination approaches to overcome osimertinib resistance (Table 1).

Multiple treatment options for osimertinib first-line progression are under investigation in the ORCHARD trial (NCT03944772), an open-label biomarker-directed phase II study with an innovative platform design. Patients enrolled underwent tumor biopsy at the time of disease progression to define the resistance mechanism and, based on biomarker analysis, were assigned to the appropriate group of treatment. Biomarker-positive patients are allocated to a biomarker-matched study treatment in group A, including the combination of osimertinib with savolitinib in case of MET amplifications, with gefitinib for C797X EGFR mutations, with necitumumab in case of EFGR amplification, and other future combinations which could be added. Patients without a biomarker are allocated to a study treatment (durvalumab + platinum-based chemotherapy, osimertinib + necitumumab, or further combinations) in group B. In group C, in the observational arm, there are patients who are not eligible for either of the previous two groups and are treated in accordance with local practice.^37^

The TRAEMOS phase I/II study (NCT03784599) is investigating the combination of osimertinib and trastuzumab–emtansine, a conjugate of the mAb trastuzumab and the cytotoxic agent DM1, which was reported to overcome osimertinib resistance in T790M-positive EGFR-mutated NSCLC cell lines that gained HER2 amplification.^73^

Similarly, combining osimertinib with drugs targeting other relevant molecular pathways, such as the Bcl-2 inhibitor APG-1252 (NCT04001777) and the poly(ADP-ribose) polymerase inhibitor niraparib (NCT03891615), represents a promising strategy under evaluation. Due to the effect of aspirin in reducing AKT phosphorylation, a combination study of osimertinib with aspirin is also ongoing (NCT03532698).

The combination of the third-generation EGFR-TKI nazartinib with the MEK inhibitor trametinib is under investigation in the phase I EATON trial (NCT03516214) both for first-line therapy of EGFR-mutated NSCLC and after the failure of previous EGFR-TKIs.

Anlotinib is a novel multitarget TKI that targets vascular endothelial growth factor receptor, fibroblast growth factor receptor, platelet-derived growth factor receptors, and c-kit. The combination of anlotinib with chemotherapy with platinum/pemetrexed is being evaluated in the phase II ALTER-L031 (NCT04136535) trial in EGFR-positive patients with disease progression to osimertinib. Furthermore, the association of anlotinib with pemetrexed and toripalimab, an anti-programmed cell death protein 1 (PD-1) inhibitor, is being assessed in a T790M-positive population after osimertinib failure (NCT04316351).

### Targeting progression with no identified mechanism of resistance

#### Osimertinib beyond progression or chemotherapy

As with early-generation EGFR-TKIs, different radiologic patterns of disease progression to osimertinib can lead to a specific therapeutic approach.^74^, ^75^, ^76^ In case of ‘oligo-progression’ with a limited number of metastatic sites and asymptomatic disease, continuing TKI therapy with or without local ablative therapy (LAT), mainly radiotherapy, is considered a valid option. Conversely, symptomatic or systemic progression with wide disease dissemination requires a change of systemic therapy.

Regarding these approaches, evidence has emerged also for osimertinib in a real-life context. In a retrospective study including two institutions from the Unites States, in 47 of 76 (61%) patients, osimertinib was continued beyond progression, achieving a median second PFS of 12.6 months and of 15.5 months in the 21 patients (44%) who received radiotherapy on sites of progression. Continuation of osimertinib beyond progression was associated with a longer OS compared with discontinuation (11.2 versus 6.1 months, *P* = 0.02).^77^

Another multi-institutional retrospective study conducted in Italy enrolled 144 patients. This study showed that, among 91 patients receiving at least one subsequent treatment, 50 (54.9%) patients (who continued osimertinib with and without LAT) achieved longer post-progression PFS (6.4 versus 4.7 months, *P* = 0.0239) and OS (11.3 versus 7.8 months, *P* = 0.0446) compared with patients who switched therapy. Among patients maintaining osimertinib, better outcomes were observed if LAT was associated.^78^

Despite many limitations regarding the small sample size and the potential selection bias related to the retrospective design, these studies confirmed that, in case of ‘non-druggable’ disease progression, maintaining osimertinib beyond progression (with adjunctive LAT) is a reasonable and effective option to be considered.

When a switch of therapy is required, the most used treatment in clinical practice is chemotherapy, namely a platinum-doublet regimen in fit patients. The analysis of post-osimertinib treatments in the FLAURA trial confirmed these data. Among patients randomized to osimertinib who received a subsequent treatment, about two-thirds (68%) received chemotherapy. A retrospective study in a real-world setting showed only a trend for longer OS in patients (20 of 65, 30.8%) who received subsequent chemotherapy, compared with those receiving a non-chemotherapy regimen (25.0 versus 11.8 months, *P* = 0.106) after progression to osimertinib. In the small subgroup of patients (21 cases) with rapid and symptomatic progression, OS and post-progression OS were significantly longer in patients who received chemotherapy than in those treated with a non-chemotherapy regimen (median OS, 12.9 versus 7.5 months, *P* =  0.006; median post-progression OS, 8 versus 1.1 months, *P* <  0.001).^77^

When histologic transformation to SCLC occurs, a platinum/etoposide regimen represents a valid option. In a retrospective study on 58 patients who developed high-grade neuroendocrine carcinomas after at least one previous EKGR-TKI, including osimertinib (18 of 58, 31%), treatment with platinum/etoposide showed a median OS of 10.9 months since the time of SCLC transformation, which was similar to that observed in the case of *de novo* SCLC receiving chemotherapy.^79^ In this study, platinum/etoposide and taxanes were the most used regimens, and interestingly no responses were observed when immune checkpoint inhibitors (ICIs) were used.

#### Combination of osimertinib with chemotherapy

Current data suggest that first-generation EGFR-TKIs erlotinib and gefitinib are well tolerated when they are combined with standard chemotherapy.^80^^,^^81^ However, results from the IMPRESS trial did not demonstrate a clinical benefit of continuing gefitinib with chemotherapy after disease progression.^82^ On the other hand, recent results of a phase III trial, the NEJ009 study, showed that the combination of gefitinib with chemotherapy improved PFS and OS in untreated patients with an acceptable toxicity profile compared with gefitinib alone, although the OS benefit requires further validation because it was derived from exploratory analysis.^83^

Preliminary results showed that the addition of platinum-based chemotherapy to osimertinib therapy is well tolerated in patients pretreated with earlier-generation EGFR-TKIs.^84^ To date, the only available data on combination chemotherapy and osimertinib in the context of osimertinib resistance are derived from experiences of off-label use.

In a retrospective study, outcomes of 44 patients with metastatic EGFR-mutated NSCLC who received osimertinib plus chemotherapy, including platinum doublets or single agents, as second-line or later therapy, were analyzed. Nearly all patients had previously received single-agent osimertinib (98%) and CNS metastases were present at baseline in most cases (86%). Osimertinib was given at 80 mg daily, 160 mg daily, or 80 mg every other day. The combination treatment did not significantly increase toxicities, even though higher rates of thrombocytopenia (59%, any grade) and neutropenia (31%, any grade) were noted than in historical chemotherapy controls. The median duration of treatment (mDOT) was 5.3 months [95% confidence interval (CI) 3.6-8 months] in the overall population, in particular 6.1 months [95% CI 4.1 months-not reached (NR)] in patients receiving platinum-doublet chemotherapy and 3 months (95% CI 1.8-4.8 months) among those who received single-agent chemotherapy. These results are comparable with historical controls, but CNS disease control was better than expected for chemotherapy alone, with low rates of CNS progression (24%).^85^

Piotrowska et al. conducted another retrospective analysis evaluating concurrent combination of osimertinib plus different chemotherapy regimens in 18 patients with EGFR-mutated NSCLC who were heavily pretreated (median of 3 lines of therapy); 16 of them were T790M positive and all had progressed on single-agent third-generation EGFR-TKI before the addition of chemotherapy. In this small cohort, mDOT on platinum doublet/osimertinib was 6.9 months (95% CI 1.4 months-NR) and 4.3 months (95% CI 1.3 months-NR) on pemetrexed/osimertinib. Clinically significant CNS progression (requiring radiation or palliative care) was observed in very few cases, all of them with previous CNS involvement. Osimertinib did not appear to add significant toxicity, and mDOT on platinum doublet/osimertinib compared favorably with the median PFS with platinum/pemetrexed in AURA3. Most patients without brain metastases at baseline did not progress in the CNS.^86^

A prospective study of carboplatin/pemetrexed/osimertinib in patients with systemic progression on osimertinib is planned to quantify CNS protection (PROTECT trial). Further data regarding the combination of osimertinib with platinum-based chemotherapy as first-line therapy will derive from the currently ongoing FLAURA 2 trial (NCT04035486).

#### Immune checkpoint inhibitors

Anti-PD-1 and programmed death-ligand 1 (PD-L1) mAbs, which have radically changed the current treatment scenario for NSCLC, seem ineffective in *EGFR*-mutated patients. The lack of benefit in patients harboring tumors with both *EGFR* mutations and *ALK* rearrangements was demonstrated in early phase III trials evaluating single-agent ICI versus docetaxel,^87^, ^88^, ^89^ with a retrospective study and meta-analyses confirming these findings.^90^^,^^91^ The expression level of PD-L1 is recognized as a predictive biomarker of efficacy for PD-L1 inhibitors. Preclinical studies demonstrated PD-L1 overexpression by EGFR-mutated tumor cells as a mechanism of immune escape.^92^ The available clinical data are discordant with regard to PD-L1 expression in EGFR-mutated NSCLC,^93^^,^^94^ with two meta-analyses failing to confirm a positive correlation with EGFR mutations, probably for differences in the types of specimens analyzed and in the PD-L1 testing.^95^^,^^96^ The phase II ATLANTIC study showed a modest activity of durvalumab (anti-PD-L1 mAb) in a cohort of heavily pretreated patients (at least two previous lines of therapy) with *EGFR*-mutated NSCLC, with a higher RR in tumors with PD-L1 expression level ≥25%.^97^ Another phase II trial evaluating pembrolizumab in TKI-naive *EGFR*-mutated patients was prematurely closed for evidence of no response after the enrollment of 11 patients, despite very high levels of PD-L1 expression (73%).^98^ Overall, the evidence is against the use of single-agent checkpoint inhibitors in both treatment-naive and TKI-pretreated *EGFR*-mutated patients, despite PD-L1 expression. Possible explanations for the poor effect of PD-L1 inhibitors in EGFR-mutated NSCLC could be the small number of CD8^+^ tumor-infiltrating lymphocytes and the low tumor mutational burden of these tumors, which is associated with a limited number of neoantigens and thus a lower likelihood of response to ICIs.^99^^,^^100^ Combination of ICI with other agents has been explored as a strategy to improve the efficacy of immunotherapy in *EGFR*-mutated NSCLC. In the phase IB multi-arm TATTON trial, the combination of osimertinib and the ICI durvalumab showed encouraging efficacy results, both in TKI-pretreated and TKI-naive patients, but enrollment into this arm has been stopped owing to an increase of pulmonary toxicity.^65^

So far, the only positive clinical data on efficacy of immunotherapy for both *EGFR*-mutated and *ALK*-rearranged tumors are derived from the phase III IMpower150 trial, which combined PD-L1 inhibition, chemotherapy, and bevacizumab.^101^ Bevacizumab alone or in combination with chemotherapy can promote T-cell tumor infiltration through tumor vasculature normalization and a decrease of IL-6. In this way, the ‘cold’ tumor microenvironment (typically seen in EGFR-mutated tumors) may be turned into an environment enriched with tumor-infiltrating lymphocytes.^102^ Moreover, EGFR activation has been shown to promote vascular endothelial growth factor expression, which might enhance the sensitivity of patients with *EGFR* mutations to anti-angiogenic drugs.^103^ Bevacizumab in combination with erlotinib improved clinical benefit compared with erlotinib alone in patients with activating EGFR mutations.^104^ The IMpower150 trial randomized chemotherapy-naive patients with advanced non-squamous NSCLC, including patients with *EGFR* or *ALK* genetic alterations who had disease progression or were intolerant to at least one line of therapy with an approved TKI (10% of the overall trial population), to one of three groups: atezolizumab/carboplatin/paclitaxel (ACP), carboplatin/paclitaxel/bevacizumab (BCP), or atezolizumab/carboplatin/paclitaxel/bevacizumab (ABCP). PFS and OS were the co-primary endpoints of the study.

Significant improvements in both endpoints with ABCP versus BCP were observed in the intention-to-treat wild-type population, and the safety profile of the ABCP combination was shown to be consistent with the safety profiles of the individual drugs.^90^^,^^105^ Subgroup analysis revealed a significant OS and PFS benefit in patients with sensitizing *EGFR* mutations and in those who received an EGFR-TKI therapy, even if in this latter group the hazard ratio and CI for OS crossed the boundary. No significant difference in survival outcomes was noted between the ACP and BCP arms, confirming that only the combination of atezolizumab and bevacizumab added to chemotherapy could provide benefit in this patient population.^106^

Only one patient in the ABCP group and five in the BCP group had previously received osimertinib. Thus, it is impossible to make a conclusive statement of the efficacy of the quadruplet therapy in the specific context of osimertinib failure. Besides the major limitation of small sample size, the subgroup analysis of EGFR-mutated patients, even if pre-specified, should be considered exploratory from a rigorous statistical point of view, and therefore not sufficiently powered to detect differences between treatment regimens. Imbalances resulted in mutation type, smoking history, and previous TKI use. Regarding safety, serious adverse events occurred in 64% of *EGFR*-mutated patients who received ABCP. The toxicity profile, in addition to the frailty of certain heavily pretreated patients, could raise some concerns on the tolerability of the quadruplet regimen in this particular setting. However, based on these data and probably driven by the need for novel treatment options, regulatory authorities have approved atezolizumab in combination with bevacizumab, paclitaxel, and carboplatin for patients with metastatic NSCLC and *EGFR/ALK* alterations after failure of approved targeted therapy.

According to the results from IMpower150, also in the IMpower130 trial,^107^ which assessed carboplatin and nab-paclitaxel with or without atezolizumab, EGFR-mutated patients did not derive benefit from the simple addition of the PD-L1 inhibitor to platinum-based chemotherapy. Even in this study, the analysis in this subgroup of patients was exploratory only. The role of combination of chemotherapy with immunotherapy could be definitively clarified by the phase III KEYNOTE 789 trial (NCT03515837), which is currently investigating the efficacy and safety of pembrolizumab added to cisplatin or carboplatin plus pemetrexed, specifically in EGFR-mutated patients after the failure of a previous TKI, including osimertinib.

### Proposed approach to manage NSCLC progression on osimertinib

**Figure 2:**
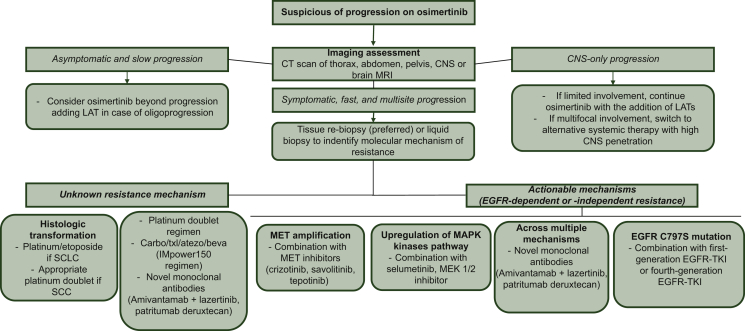
Proposed algorithm for managing epidermal growth factor receptor (EGFR)-mutated non-small-cell lung cancer progressing on osimertinib. CNS, central nervous system; CT, computed tomography; LAT, locally ablative therapy; MRI, magnetic resonance imaging; SCC, squamous cell carcinoma; SCLC, small-cell lung cancer; TKI, tyrosine kinase inhibitor.

Based on the above-mentioned data, we proposed an algorithm for managing EGFR-mutated NSCLC progressing on osimertinib (Figure 2).

In case of clinical suspicion of progression to osimertinib, complete imaging of the chest, abdomen, pelvis, and CNS is recommended. When progression is asymptomatic and the disease is slowly growing, continuing osimertinib beyond progression is a reasonable option in addition to strict clinical and radiologic monitoring of disease. The association of LAT on oligo-progressive sites of disease may improve the outcome. Also, patients with limited CNS progression should continue osimertinib when receiving local therapy (stereotactic radiotherapy or neurosurgery in selected cases). If CNS involvement is wide and symptomatic, switching to a different systemic therapy with high intracranial penetration (such as chemotherapy with carboplatin/pemetrexed) as an alternative or in addition to local therapy (namely whole-brain irradiation) should be advisable.

In case of wide, fast, or symptomatic progression, switching of systemic therapy is mandatory. Enrollment of patients in clinical trials is encouraged. We strongly recommend to investigate the potential molecular mechanisms of resistance with liquid biopsy or tissue re-biopsy. Nevertheless, tissue biopsy is the only way to identify histologic transformation that requires appropriate chemotherapy treatment; moreover, liquid biopsy is also flawed by its limits of sensitivity because not all cancers shed enough DNA to be detected. In the landscape of progression disease to osimertinib, relevant treatment strategies directed against a specific molecular resistance mechanism have been developed, such as combination with first-generation EGFR-TKI, MET, or MEK inhibitors and the use of novel mAbs active across multiple resistance pathways or a novel fourth-generation EGFR-TKI able to bypass EGFR resistance mutations. Novel mAbs seem to be active also when a specific resistance mechanism cannot be identified.

In cases where the resistance mechanisms remain unknown or histological transformation occurred and enrollment in clinical trial is not available, chemotherapy including a platinum doublet represents a feasible option in patients maintaining a good performance status. Single-agent immunotherapy, even if more tolerable than cytotoxic agents, is not active in *EGFR*-mutated NSCLC. Although the combination of osimertinib with durvalumab showed an interesting activity in patients pretreated with TKI, the observed lung toxicity limits this approach. Combination treatment of carboplatin/paclitaxel with atezolizumab and bevacizumab prolonged survival in patients who progressed on previous EGFR therapy, but limited data are available on an osimertinib-treated population and there are some concerns about the safety of this quadruplet in real-life population.

## Conclusions

The treatment options following resistance to osimertinib are rapidly changing, moving from the inevitable recourse to chemotherapy (when clinically feasible) to various treatment strategies, including targeted drugs or novel combination approaches. The future challenge is represented by the discovery of novel resistance pathways using more sophisticated methods, such as in-depth and genome-wide DNA, RNA, and protein expression analyses, to increase the potentiality of developing targeted treatments. Trials with an innovative platform design, such as the ORCHARD trial, in which patients are allocated to a biomarker-matched study treatment based on their molecular profile, will hopefully allow clinicians to apply an effective personalized approach in this growing patient population.
